# Ambient pH Controls Glycogen Levels by Regulating Glycogen Synthase Gene Expression in *Neurospora crassa*. New Insights into the pH Signaling Pathway

**DOI:** 10.1371/journal.pone.0044258

**Published:** 2012-08-31

**Authors:** Fernanda Barbosa Cupertino, Fernanda Zanolli Freitas, Renato Magalhães de Paula, Maria Célia Bertolini

**Affiliations:** 1 Departamento de Bioquímica e Tecnologia Química, Instituto de Química, Universidade Estadual Paulista, UNESP, Araraquara, São Paulo, Brazil; 2 Nalco Company, Sugar Land, Texas, United States of America; University of Minnesota, United States of America

## Abstract

Glycogen is a polysaccharide widely distributed in microorganisms and animal cells and its metabolism is under intricate regulation. Its accumulation in a specific situation results from the balance between glycogen synthase and glycogen phosphorylase activities that control synthesis and degradation, respectively. These enzymes are highly regulated at transcriptional and post-translational levels. The existence of a DNA motif for the *Aspergillus nidulans* pH responsive transcription factor PacC in the promoter of the gene encoding glycogen synthase (*gsn*) in *Neurospora crassa* prompted us to investigate whether this transcription factor regulates glycogen accumulation. Transcription factors such as PacC in *A. nidulans* and Rim101p in *Saccharomyces cerevisiae* play a role in the signaling pathway that mediates adaptation to ambient pH by inducing the expression of alkaline genes and repressing acidic genes. We showed here that at pH 7.8 *pacC* was over-expressed and *gsn* was down-regulated in wild-type *N. crassa* coinciding with low glycogen accumulation. In the *pacC^KO^* strain the glycogen levels and *gsn* expression at alkaline pH were, respectively, similar to and higher than the wild-type strain at normal pH (5.8). These results characterize *gsn* as an acidic gene and suggest a regulatory role for PACC in *gsn* expression. The truncated recombinant protein, containing the DNA-binding domain specifically bound to a *gsn* DNA fragment containing the PacC motif. DNA-protein complexes were observed with extracts from cells grown at normal and alkaline pH and confirmed by ChIP-PCR analysis. The PACC present in these extracts showed equal molecular mass, indicating that the protein is already processed at normal pH, in contrast to *A. nidulans.* Together, these results show that the pH signaling pathway controls glycogen accumulation by regulating *gsn* expression and suggest the existence of a different mechanism for PACC activation in *N. crassa.*

## Introduction

*Neurospora crassa* is a filamentous fungus that has been widely used as a model organism for studies on fundamental aspects of eukaryotic biology. The completion of its genome sequence [1] and, more recently, the availability of a set of mutant strains individually knocked-out in specific genes have enormously accelerated the investigation of many aspects of the biology of this organism. We have been using *N. crassa* to study how the metabolism of glycogen, a storage carbohydrate that functions as carbon and energy reserve, is regulated. Glycogen biosynthesis starts with the self-glucosylating initiator enzyme glycogenin (EC 2.4.1.186), termed GNN in *N. crassa* [2]. After an oligosaccharide is synthesized, the elongation step is catalyzed by glycogen synthase (EC 2.4.1.11) and a branching enzyme (EC 2.4.1.18) that mediate the formation of α-1,4- and α-1,6-glycosidic bonds, respectively, characteristic of the glycogen molecule. Glycogen synthase is the rate-limiting enzyme in this process and is inhibited by phosphorylation; however, the phosphorylated form can be activated by the allosteric effector glucose-6-phosphate [3].

In *N. crassa,* glycogen synthase (GSN) has been characterized at gene and protein levels. Under normal growth conditions, *gsn* expression is maximal at the end of the exponential growth phase, which coincides with the highest glycogen content. However, under stress conditions, such as heat shock, *gsn* expression is down-regulated and the enzyme is less active. This may explain the decrease in glycogen levels observed in cells exposed to heat shock [4]–[6]. *gsn* expression and GSN phosphorylation are also regulated by the cAMP-protein kinase A (PKA) signaling pathway [7]. Thus, the regulation of glycogen metabolism in *N. crassa* involves different proteins whose activation may be influenced by different signaling pathways. Analysis *in silico* of the *gsn* 5′-flanking region led to the identification of *cis* DNA-binding motifs specific for transcription factors acting via different signaling pathways [8]. These motifs include a recognition site for the *A. nidulans* zinc-finger transcription factor PacC, suggesting a possible role for pH in regulating glycogen synthesis.

PacC has been extensively studied in *A. nidulans* and plays an important role in the pH signaling pathway. This transcription factor mediates the cell adaptation to neutral-alkaline pH by activating genes that are preferentially expressed at alkaline pH and repressing those preferentially expressed at acidic pH [9]. In *A. nidulans*, PacC is activated by two successive proteolytic cleavage steps at the C-terminus, the first being pH-dependent and activated by the products of six *pal* genes while the second is proteasome-mediated and pH-independent [10], [11]. The proteolysis lead to the active protein PacC^27^ that contains a DNA-binding domain formed by three C_2_H_2_ zinc fingers capable of binding to the core consensus sequence 5′-GCCARG-3′ present in the promoters of pH-regulated genes (reviewed in [12], [13]).

The Rim101p, a PacC orthologue in *S. cerevisiae*, was initially identified as a positive regulator of meiosis and *rim101* mutants are sensitive to Na^+^ or Li^+^ ions and grow poorly at low temperatures [14]. The yeast protein therefore appears to have a broader role than simply that of promoting alkaline pH-inducible responses [15]. While Rim101p is associated with the pH response, there are important differences between PacC and Rim101p. For example, the yeast protein requires only a single cleavage step to be activated [16] and, whereas PacC acts as a transcriptional activator under alkaline pH, Rim101p exerts its role as a repressor [15]. In addition, at least three pathways participate in the response to high pH stress in *S. cerevisiae* (reviewed in [17]). Thus, the molecular mechanisms involved in the pH response differ among organisms.

PacC orthologues have been identified in numerous filamentous fungi and the role of PacC as a mediator of pH regulation in fungal pathogenicity was first described in *Candida albicans,* in which Rim101p governs pH responses, dimorphism, and pathogenesis [18]. In *N. crassa,* the PACC pathway is involved in the glycosylation of Pi-repressible acid phosphatase [19] and in the transcription of the *hsp70* gene [20].

In this report, we demonstrated that the pH signaling pathway regulates glycogen metabolism in *N. crassa* by a process in which PACC may play a central role. As an *A. nidulans* PacC motif was identified in the *gsn* promoter, and since *N. crassa* has the six *A. nidulans pal* gene orthologues, we investigated the influence of pH on glycogen accumulation and the regulation of *gsn* expression. Gel mobility shift assays showed that recombinant PACC recognized and bound specifically to the *gsn* promoter; this binding was confirmed *in vivo* by ChIP-PCR analysis. We also found that PACC proteolysis may involve a different way from that described for the *A. nidulans* protein.

## Results

### Knockout of the *N. crassa pacC* Gene and Growth Phenotype Analysis

**Figure 1:**
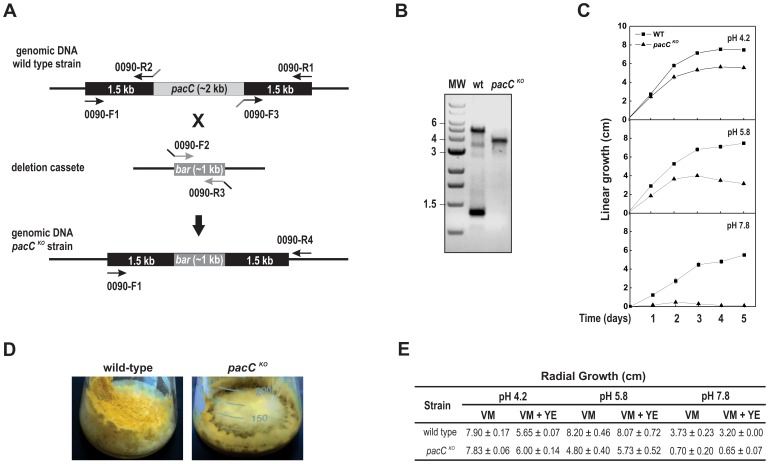
*pacC* gene knockout and phenotypic analysis of the mutant strain. (A) Schematic illustration of the *pacC* gene knockout strategy. (B) Diagnostic PCR for validation of the *pacC* knockout was performed by using 0090-F1 and 0090-R4 oligonucleotides (Table 1). (C) Linear growth analysis. Apical extension of basal hyphae was determined in race tubes, as described in Material and Methods. The results shown are the average of three independent experiments. (D) Melanization. Strains were cultured in 250 mL flasks containing VM medium for 10 days (3 days at 30°C in the dark and 7 days at room temperature in ambient light/dark). Melanization can be visualized as brown pigment formation in the *pacC^KO^* strain. (E) Radial growth analysis. Basal hyphae growth was examined after cultivating the strains on plates containing solid VM medium, as described in Material and Methods. Growth was expressed as colony diameter.

To investigate the role of PACC in the regulation of glycogen metabolism we generated a null deleted strain by replacing the entire *pacC* ORF (NCU00090, 1,866 bp) with the *bar* gene in a *mus-52^KO^* background strain (Figure 1A). The gene replacement and correct integration were confirmed by PCR analysis of the transformants (Figure 1B). However, we were unable to eliminate the *mus-52* mutation after crossing the transformants with a wild-type strain due to the close linkage between the *mus-52* and *pacC* genes. The morphological characteristics of the *pacC^KO^* strain were analyzed by growth on VM agar plates and in race tubes. Since there were no apparent differences in the phenotypes of the *mus-51^KO^* (used as the wild-type in this work) and *mus-52^KO^* strains [21] we concluded that the growth defects observed in the *pacC^KO^* strain were caused specifically by the absence of the transcription factor. Linear growth analysis in race tubes showed that both strains grew better at acid (4.2) and normal (5.8) pH than at alkaline pH and that the *pacC^KO^* strain was unable to grow at alkaline pH (7.8) (Figure 1C). We have also observed an accumulation of brown pigments after five days under normal growth conditions in flask (Figure 1D), characterized as melanin and described in other transcription factor mutant strains [22].

**Figure 2:**
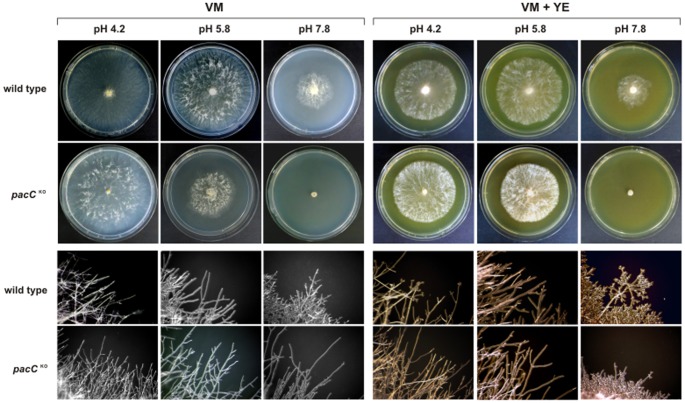
Phenotypes of the wild-type and *pacC^KO^* strains in different pH and nutritional growth conditions. The strains (10^7^ conidia/mL) were cultured on plates containing agar VM medium and VM medium supplemented with yeast extract (VM + YE) at pH 4.2, 5.8 and 7.8, in ambient light/dark for 24 h. Hyphae at the colony edge were observed using a stereoscope.

Radial growth was analyzed in plates after 24 h of growth in VM medium and VM medium supplemented with yeast extract (Figures 1E and 2). The *pacC^KO^* strain displayed similar growth to the wild-type strain in both media at acid pH and slightly reduced growth at normal pH; the *pacC^KO^* strain did not grow at alkaline pH. The strains showed enhanced hyphal branching and shorter aerial hyphae in both media at alkaline pH (Figure 2). However, the *pacC^KO^* strain displayed severe growth defects at alkaline pH, including a reduction in the extent and production of aerial hyphae. Together, these results show that PACC is required for growth under alkaline conditions.

### Glycogen Levels and Gene Expression Under pH Stress

**Figure 3:**
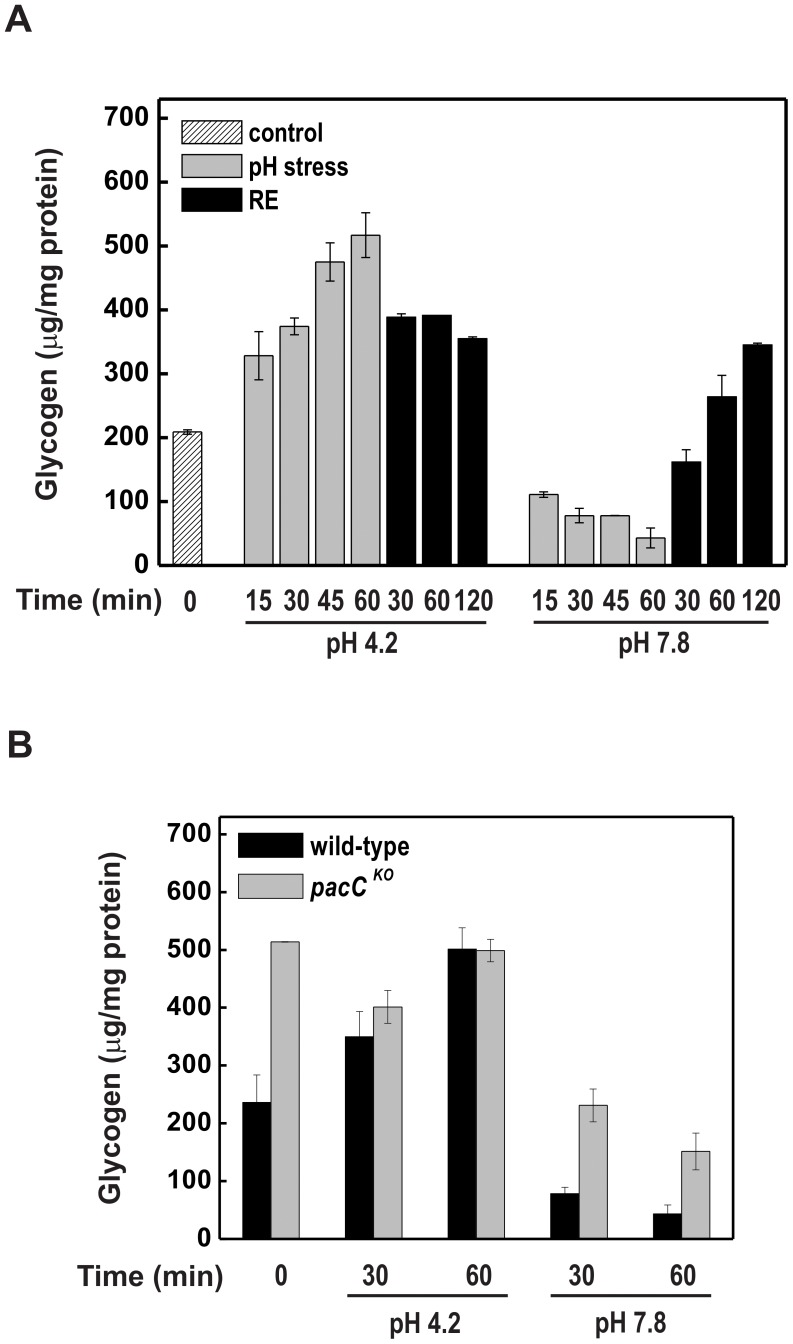
Glycogen accumulation during acid and alkaline pH stress. (A) Glycogen content in the wild-type strain. Glycogen was extracted from mycelia grown under physiological conditions (pH 5.8, control) and under acid (pH 4.2) and alkaline (pH 7.8) stress. After 120 min, the remaining cultures were transferred back to physiological conditions (pH 5.8, RE, recuperation). The results shown are the average of at least three independent experiments. (B) Glycogen content in the *pacC^KO^* strain compared to the wild-type strain. 0, cell samples before the pH shift (control).

The *gsn* gene is regulated at transcriptional and post-translational levels. We have previously reported that gene expression and glycogen levels were altered under stress conditions such as heat shock and carbon starvation [5], [6], both of which affect glycogen metabolism in *N. crassa*. The identification of a DNA motif for the *A. nidulans* pH signaling regulator PacC in the *gsn* promoter [8] prompted us to investigate whether this gene could be regulated by pH stress. Cells from both strains were germinated at pH 5.8 and then subjected to acid and alkaline pH stress after which samples were collected for glycogen quantification and RNA extraction. The glycogen levels in the *pacC^KO^* strain were higher than in the wild-type strain at normal growth pH (pH 5.8) (Figure 3B), indicating that PACC is important for maintaining normal glycogen levels during vegetative growth. However, under acid stress (pH 4.2), both strains accumulated similar amounts of glycogen (Figure 3A and 3B), suggesting that PACC does not affect glycogen accumulation under this condition in agreement with the growth results shown in Figure 1C. At alkaline pH, there was a decrease in the glycogen levels of both strains compared to the levels at pH 5.8. The fact that the glycogen level in the *pacC^KO^* strain after 30 min of alkaline stress was similar to that of the non-stressed wild-type strain (Figure 3B, time 0) emphasizes the role of PACC as a major regulator of glycogen accumulation under alkaline pH stress. The glycogen levels were also quantified in wild-type cells that were returned to normal pH after being subjected to acid and alkaline pH stress (RE, recuperation), however they did not recover the glycogen levels seen before stress (Figure 3A) in both conditions.

**Figure 4:**
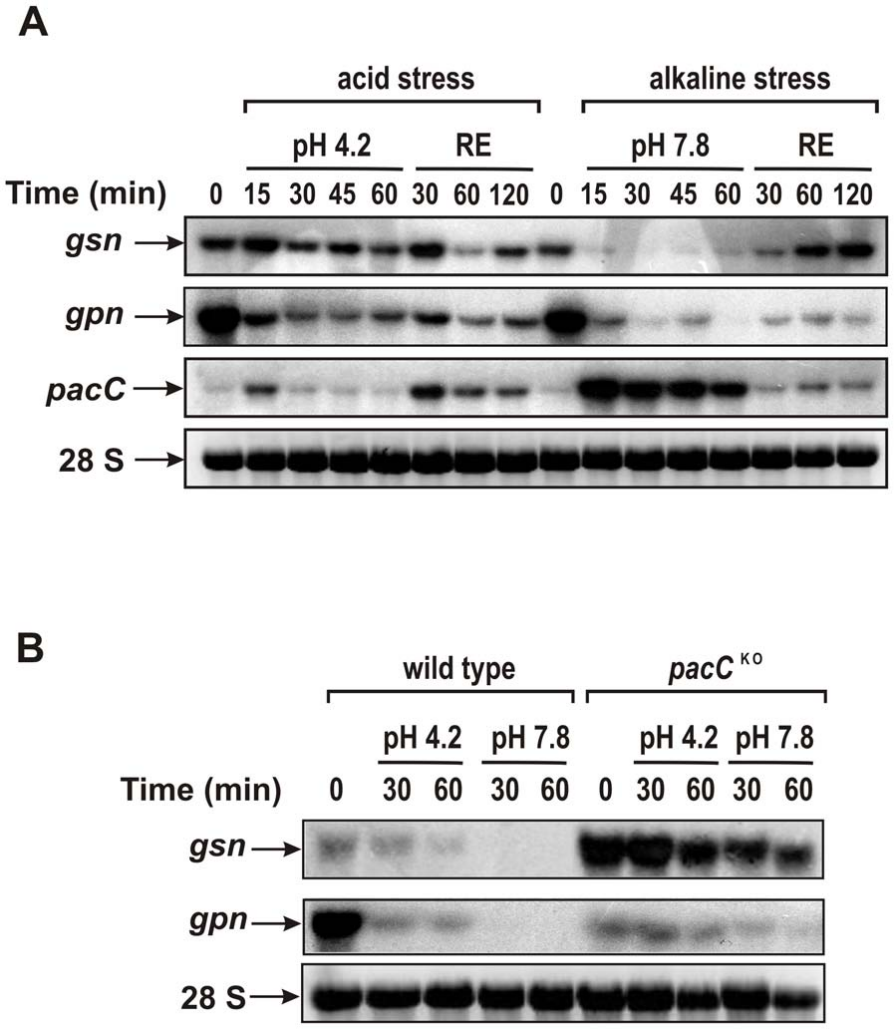
*gsn, gpn* and *pacC* gene expression during acid and alkaline pH stress. Cells from the wild-type and *pacC^KO^* strains were cultivated at pH 5.8 for 24 h and shifted to pH 4.2 and pH 7.8. Samples were collected and used to extract total RNA. Total RNA (15 µg) was separated by electrophoresis in a denaturing formaldehyde gel, transferred to nylon membrane and probed with α-^32^P-radiolabeled 678 bp *gsn* cDNA, or 798 bp *gpn* cDNA or 639 bp *pacC* cDNA fragments (gel autoradiographies). The 28 S rRNA was used as a loading control after ethidium bromide staining. The results shown are the average of at least three independent experiments. (A) Analysis of the *gsn, gpn* and *pacC* genes in the wild-type strain at different times after pH shifting. After pH stress the remaining cultures were transferred back to physiological conditions (RE, recuperation, pH 5.8) and samples were collected. (B) Analysis of the *gsn* and *gpn* genes in the *pacC^KO^* strain compared to the wild-type strain at different times after pH shifting. 0, cell samples before pH shifting (control).

To investigate whether the effect of extracellular pH on glycogen accumulation resulted from changes in *gsn* and *gpn* (named here as the gene encoding the enzyme glycogen phosphorylase) expression we analyzed their mRNA levels during pH stress and recuperation at pH 5.8 in a wild-type strain. During acidic stress, the *gsn* transcript levels were kept constant while the *gpn* transcript decreased during 60 min of incubation (Figure 4A) and the levels were not recovered after returning the cells to normal growth pH (Figure 4A, RE). When cells were transferred to alkaline medium, *gsn* and *gpn* transcript levels decreased markedly during 60 min of stress, however only *gsn* levels were recovered to normal levels after 60 min of incubation at pH 5.8 (Figure 4A). These results indicate that changes in extracellular pH control *gsn* expression. We also analyzed the *pacC* mRNA levels under pH stress and recuperation at pH 5.8 in the wild-type strain (Figure 4A). The *pacC* transcript size was ∼3 kb. Whereas *pacC* expression was low at acid pH, at alkaline pH there was a marked increase in expression compared to that at physiological pH (5.8), mainly during the first 15 min of stress. Basal expression was achieved when the mycelia were transferred back to pH 5.8 (RE). Expression of *gsn* and *gpn* genes was analyzed in the *pacC^KO^* strain under acid and alkaline pH stress and compared to the wild-type strain. The *gsn* mRNA levels were higher in the *pacC^KO^* strain than in the wild-type strain at all pH values (Figure 4B), suggesting a repressive role for PACC in *gsn* expression. This result explains, at least partially, the glycogen levels shown in Figure 3B. There was very low expression for *gpn* gene in the mutant strain in all pH suggesting that PACC also controls *gpn* expression under normal pH. Together, the results shown in Figures 3B and 4 suggest that alkaline pH induces *pacC* expression and a concomitant decrease in glycogen levels by down-regulating the *gsn* gene. These findings characterize *gsn* as an acid-specific gene.

### Glycogen Levels and Gene Expression Under Combined pH and Heat Stress

**Figure 5:**
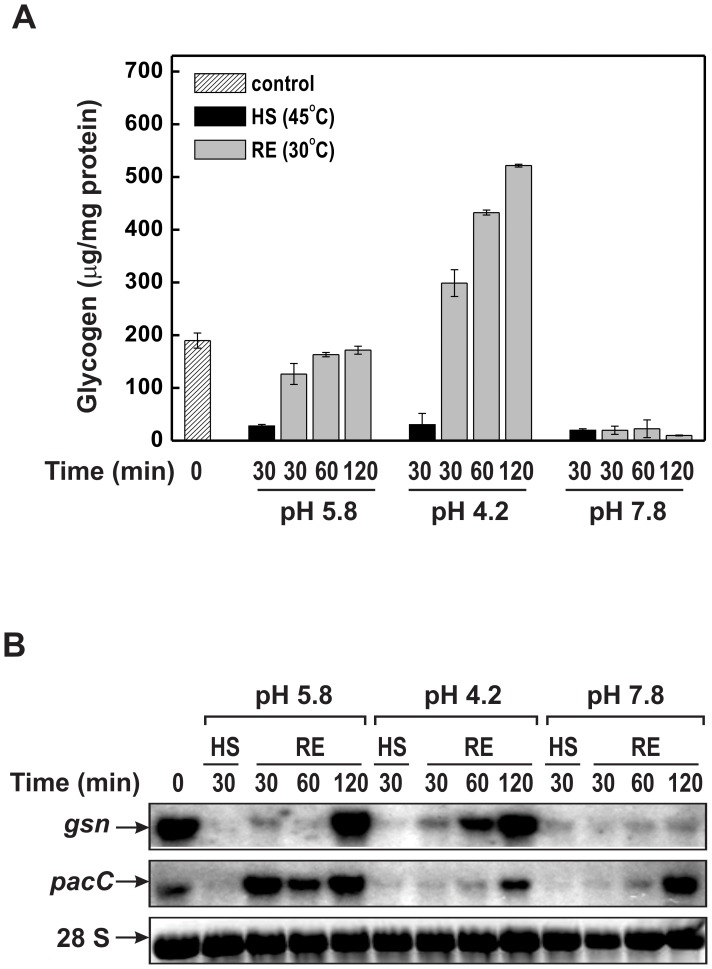
Glycogen accumulation and gene expression during combined pH and heat shock stress. Glycogen and total RNA were extracted from wild-type mycelia cultivated at pH 5.8 and 30°C for 24 h and then shifted to pH 5.8, 4.2 and 7.8 at 45°C for 30 min. After 30 min, the remaining samples were transferred back to the physiological temperature (30°C) at the three pH conditions and incubated for different times (RE, recuperation). (A) Accumulation of glycogen. (B) *gsn* and *pacC* gene expression. Total RNA (15 µg) was separated by electrophoresis in a denaturing formaldehyde gel, transferred to nylon membrane and probed with the α-^32^P radiolabeled 678 bp *gsn* cDNA and 639 bp *pacC* cDNA fragments (gel autoradiographies). The 28 S rRNA was used as a loading control after ethidium bromide staining. The results shown are the average of at least three independent experiments. 0, cell samples before pH shifting (control).

As previously described, heat shock (45°C) down-regulates *gsn* expression, resulting in decreased glycogen accumulation compared to that observed at 30°C (the normal temperature of growth) [5], [6]. We therefore examined the effect of a combination of heat and pH stress on glycogen accumulation and in the expression of *gsn* and *pacC* genes in the wild-type strain. For this, cells were germinated at pH 5.8 and transferred to media of different pH preheated to 45°C. Glycogen levels were strongly reduced during heat shock, regardless of the pH (Figure 5A, black bars, HS). When heat stress was removed while maintaining the pH stress, normal glycogen levels were regained only in media with normal and acid pH (Figure 5A, compare with the results shown in Figure 3A for pH 4.2). The cells were unable to recover normal glycogen levels at alkaline pH (Figure 5A, pH 7.8) and they were the same as under heat stress.

The influence of pH and temperature stress on *gsn* expression was also analyzed, as an attempt to correlate with glycogen accumulation. The *gsn* transcript levels were reduced in cells subjected to heat shock, regardless the media pH (Figure 5B). However, when cells were transferred back to normal temperature (30°C) and pH stress was maintained the *gsn* expression levels were recovered only at pH 4.2 and 5.8, but not at pH 7.8, in agreement with the results shown in Figure 5A. As with *gsn*, *pacC* expression was strongly repressed by heat stress at both pH values. In addition, gene expression was recovered after returning the cells to normal temperature (30°C) at all pH values (Figure 5B). This interesting result suggests that the heat signaling pathway apparently elicits stronger cellular responses than the pH signaling pathway, at least for *pacC* expression. It is noteworthy the existence of six STRE (STress Response Elements) and four HSE (Heat Shock Elements) motifs in the *pacC* promoter, all of which are DNA elements known to be responsive to heat stress.

### Recombinant PACC Binds to the *gsn* Promoter

**Figure 6:**
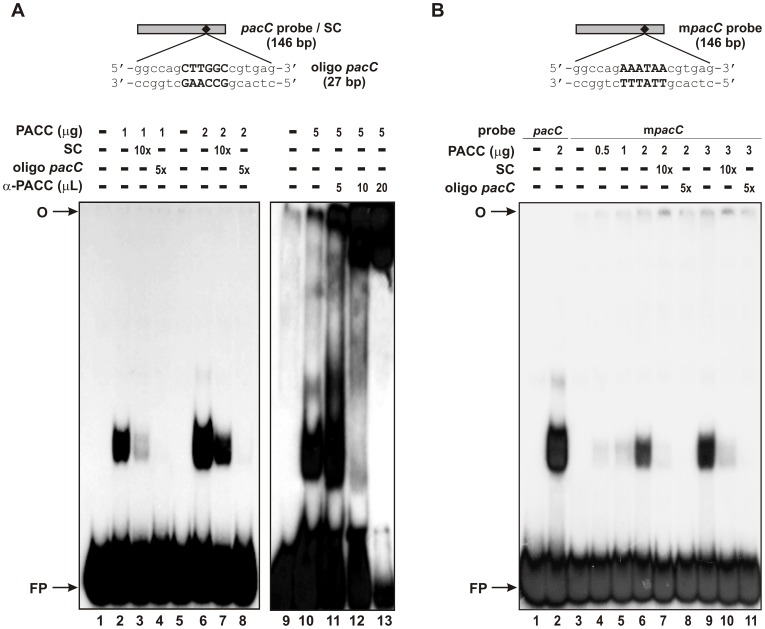
Binding of recombinant PACC to the *gsn* promoter. (A) Upper panel, schematic representation of the *pacC* probe and the specific competitor oligo *pacC*. Lower panels, gel shift analysis using increasing amounts of recombinant PACC in the presence of specific competitors and polyclonal anti-PACC antibody. Lanes 1, 5 and 9, *pacC* probe, no protein added. Lanes 2, 6 and 10, gel shift analysis using 1.0, 2.0 and 5.0 µg of recombinant PACC. Lanes 3 and 7, gel shift analysis in the presence of the 146 bp specific competitor. Lanes 4 and 8, gel shift analysis using the specific competitor oligo *pacC*. Lanes 11 to 13, supershift assay using 5.0, 10.0 and 20.0 µL of anti-PACC antibody (1∶500). (B) Upper panel, schematic representation of the mutated m*pacC* probe. Lower panel, gel shift with wild-type and mutated probes. Analysis using different concentrations of recombinant PACC in the presence and in the absence of competitors. Lane 1 and 3, *pacC* and m*pacC* probes, respectively, no protein added. Lanes O, gel origin; SC, specific competitor; FP, free probe.

To investigate whether the PacC DNA motif identified in the *gsn* promoter was, indeed, recognized by PACC, a gel mobility shift assay was performed using a truncated recombinant PACC and a 146 bp DNA fragment containing the PacC core sequence (5′-CTTGGC-3′) as probe. Recombinant PACC bound efficiently to the fragment corresponding to the PacC recognition motif in the *gsn* promoter (Figure 6A, lanes 2 and 6). DNA-protein binding was reduced in the presence of unlabelled specific competitor (Figure 6A, lanes 3 and 7). The specificity of the DNA-protein complexes was further confirmed by adding a 27 bp DNA oligonucleotide (oligo *pacC*) as a specific competitor (Figure 6A, lanes 4 and 8) and by supershift experiments (Figure 6A, lanes 11 to 13). An interesting result was the presence of two complexes exhibiting different molecular masses when the specific competitor was added to the reaction (Figure 6A, lanes 3 and 7). We speculated that they may represent complexes with distinct conformational structures. Additional experiments will be necessary to clarify this. The DNA oligonucleotide completely abolished both complexes and the addition of anti-PACC antibody to the reaction increased the molecular mass of the DNA-protein complex, which shifted close to the gel origin (Figure 6A, lanes 12 and 13).

The specificity of the DNA-protein complex was also confirmed by using a mutated *pacC* probe (m*pacC*) in which the 5′-CTTGGC-3′ core sequence was changed to 5′-AAATTA-3′ (Figure 6B). There was a marked reduction in the formation of DNA-protein complex and a weaker complex was only visible after adding 2.0 µg of protein (Figure 6B, lane 6), i.e., an amount of protein sufficient to produce a strong complex (compare with lane 2). The complex formation was strongly decreased in the presence of the specific competitor (Figure 6B, lane 7 and 10) and completely inhibited in the presence of the DNA oligonucleotide (Figure 6B, lane 8 and 11).

### Binding of Proteins from Crude Cellular Extract to the PacC Binding Site of the *gsn* Promoter

**Figure 7:**
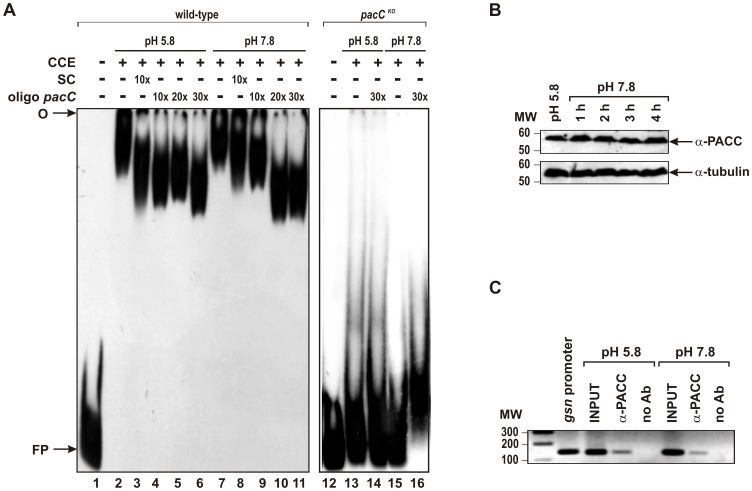
PACC binds specifically to the *gsn* promoter region in an alkaline pH-independent manner. (A) Gel shift analysis using crude cellular extracts fractionated on a Heparin-Sepharose column. Crude cellular extracts (CCE) from mycelia submitted or not to alkaline pH stress (pH 7.8) were fractionated by affinity chromatography. Left panel, a protein fraction (35 µg) exhibiting DNA-binding activity was assayed in the presence of specific competitors. Lane 1, *pacC* probe, no protein added. Lanes 2 and 7, proteins from pH 5.8 and pH 7.8 samples, respectively, in the absence of competitors. Lanes 3 and 8, DNA band shift in the presence of the 146 bp *pacC* specific competitor. Lanes 4 to 6 and 9 to 11, DNA band shifts in the presence of increasing amounts of the 27 bp DNA oligo *pacC* as a specific competitor. Right panel, a protein fraction (35 µg) from knocked-out strain crude cellular extract was assayed. Lane 12, *pacC* probe, no protein added. O, gel origin; SC, specific competitor; FP, free probe. (B) PACC shows the same molecular mass at pH 5.8 and pH 7.8. Crude cellular extracts prepared from wild-type *N. crassa* submitted or not to alkaline pH stress were analyzed by Western blotting using a polyclonal anti-PACC antibody. The protein α-tubulin (theoretical molecular mass 50 kD) was used as a loading control. (C) Chromatin immunoprecipitation assay using the polyclonal anti-PACC antibody. Genomic DNA samples from wild-type *N. crassa* submitted or not to pH stress were immunoprecipitated with the anti-PACC antibody and subjected to PCR to amplify a 146 bp DNA fragment of the *gsn* promoter containing the *pacC* motif. A plasmid construction containing the entire sequence of the *gsn* gene, including its 5′- and 3′-flanking regions, was used as a positive control. As a negative control, the immunoprecipitation reactions were performed without the anti-PACC antibody. L, 1 kb DNA ladder.

To investigate whether PACC binding to the *gsn* promoter was pH-dependent, gel shift assays were performed with crude cellular extracts from wild-type and *pacC^KO^* cells grown under normal (5.8) and alkaline (7.8) pH. The cellular extracts were fractionated by affinity chromatography, bound proteins were eluted with a KCl gradient and the fractions were analyzed for their ability to bind to the *pacC* probe previously used. Fractions from wild-type strain mycelium showing DNA-binding activities were identified in extracts from cells grown at both pH conditions (results not shown). One active chromatographic fraction from each pH was assayed for its binding to the PacC consensus site of the *gsn* promoter in the presence of both competitors. DNA-protein complexes were observed when proteins were incubated with the DNA fragment (Figure 7A, lanes 2 and 7). When the same fragment was used as a specific competitor in 10-fold molar excess the complexes were shifted only when crude cellular extract prepared from mycelia grown at pH 5.8 was used as the protein source (Figure 7A, lane 3). However, the specific competitor oligo *pacC* was able to shift the DNA-protein complexes when proteins from both extracts were analyzed (Figure 7A, lanes 4–6, 10 and 11). The binding reaction was apparently stronger when cellular extract prepared from cells grown at alkaline pH was used as a protein source since the DNA-protein complex was shifted only at higher oligo *pacC* concentrations (20- and 30-fold molar excess). Fractions from *pacC^KO^* strain were also assayed for binding reactions and no DNA-protein complex was observed. The Figure 7A (lanes 13–16) shows the results with one chromatographic fraction from each pH and the competition with the oligo *pacC*. This result indicated the existence of an active PACC protein in the wild-type strain that specifically bound to the PacC motif even under normal growth pH (5.8). It is noteworthy that the DNA-protein complexes migrated close to the gel origin suggesting that they may include other proteins in addition of PACC. It is also interesting since low levels of *pacC* transcript could be detected in wild-type cells grown at pH 5.8 (Figure 4A).

We also investigated whether the PACC present in the two cellular extracts had equal molecular mass since the same protein from *A. nidulans* grown at alkaline pH has a lower molecular mass than that observed at normal growth pH [10]. The presence of PACC in the cellular extracts was analyzed using polyclonal anti-PACC antibody. The protein was detected in both cellular extracts and, surprisingly, had the same molecular mass (<60 kD), which was similar to that of the intermediate PacC form from *A. nidulans* grown at alkaline pH (53 kD) (Figure 7B). A single PACC form was identified in the cellular extract prepared up to 4 h after the cells were shifted to alkaline pH. There was little protein induction in extracts from 1 h after shifting. The processed PacC^27^ protein described in *A. nidulans* grown at alkaline pH was not detected in *N. crassa.*

Chromatin immunoprecipitation (ChIP-PCR) experiments in the wild-type strain were performed to confirm that *N. crassa* PACC binds to the *gsn* promoter *in vivo* and to assess whether protein binding was pH-dependent. Figure 7C shows that endogenous PACC was associated with the *gsn* promoter region containing the PacC motif. In addition, protein association was observed with genomic DNA extracted from cells grown at normal pH (5.8) and from cells 2 h after shifting to alkaline pH. Together, these results indicate that *N. crassa* PACC specifically associates with the *gsn* promoter even at pH 5.8, the pH for normal growth, thus confirming that the protein is active at physiological pH.

## Discussion

Extracellular pH has an important role in cell biology as it regulates gene expression and consequently influences a variety of cellular processes, such as growth, differentiation and development. The regulation of gene expression by pH has been widely investigated in microorganisms because of the great variations in ambient pH that they face in their natural environments. The ambient pH signaling pathway has been exhaustively studied in the filamentous fungus *A. nidulans* and in several yeasts, including *S. cerevisiae.* In *A. nidulans*, pH regulation is mediated by the action of a cascade of proteins (*pal* gene products) that leads to activation of the transcription factor PacC. This protein, which has a central role in the pH signaling pathway, is activated at alkaline pH after two proteolytic cleavage steps and acts as a transcriptional activator of alkaline-expressed genes (reviewed in [23]).

In *S. cerevisiae*, the pathway leading to activation of the Rim101p orthologue shares similarities with that of *A. nidulans* PacC. However the mechanism of alkaline pH adaptation is not exactly the same since Rim101p requires only a single cleavage step to be activated and, whereas PacC acts either as an activator or repressor, Rim101p is a repressor of the genes encoding the transcriptional repressors Nrg1p and Smp1p at alkaline pH. Nrg1p is required for the repression of alkaline pH-inducible genes, including the gene encoding Ena1 Na^+^-ATPase, a protein essential for growth at alkaline pH [15]. Thus, Rim101p is indirectly required for *S. cerevisiae* adaptation to alkaline stress. In addition to Rim101p-mediated alkaline pH regulation, there are also Rim101p-independent mechanisms involved in pH regulation in *S. cerevisiae.* One is the calcineurin pathway, the activation of which affects the expression of a number of genes through dephosphorylation of the transcription factor Crz1 [24]. High pH stress triggers a transient rise in cytoplasmic calcium resulting in calcineurin activation and *ENA1* induction through Crz1 [25]. More recently, Platara *et al.* [26] described another signaling pathway involving the protein kinase Snf1p that acts through the Mig2p repressor, i.e., a third regulatory pathway involved in the response to high pH stress.

The results described here indicate a role for *N. crassa* PACC as a regulator of glycogen metabolism through its ability to target GSN via a PacC binding site in the *gsn* promoter. The *pacC^KO^* strain showed impaired growth at alkaline pH, in agreement with the better growth of *N. crassa* at acidic (5.8) compared to alkaline (7.8) pH. Our findings suggest the existence of a different mechanism of PACC activation under alkaline pH stress when compared to *A. nidulans* and *S. cerevisiae*.

### PACC Regulates Glycogen Levels Under pH Alkaline Stress through Regulation of *gsn* Expression

We investigated the glycogen levels and *gsn* expression in response to extracellular pH changes. Our results provide evidence that both processes are under pH control in *N. crassa*. Cells of the wild-type strain grown at alkaline pH showed low intracellular glycogen accumulation and a reduction in *gsn* transcript levels. In contrast, the *pacC^KO^* strain accumulated similar amounts of glycogen when compared to the non-stressed wild-type strain and showed high *gsn* transcript levels at alkaline pH. These results are sufficient to characterize *gsn* as a pH-regulated gene, and that it is an acid-specific gene. Many genes have been reported to be regulated by ambient pH [27]. In the case of *gsn*, the finding that wild-type and *pacC^KO^* strains had similar amounts of glycogen at normal and alkaline pH, respectively, reinforced the criteria for a gene to be regulated by ambient pH [28]. The analysis of *pacC* gene expression in *N. crassa* showed that it was under pH control, as also described for *A. nidulans* [9] and other filamentous fungi [29]–[31]. This observation supports the fact that, at alkaline pH, the over-expression of *pacC* controls glycogen levels by regulating *gsn*, but not *gpn*, expression.

The patterns of glycogen accumulation and gene expression under combined pH and heat stress were very interesting. Whereas in the wild-type strain glycogen and *gsn* transcript levels returned to normal, this strain was unable to recover normal levels when exposed concomitantly to heat and alkaline stress. In addition, *pacC* was not induced under these conditions. The presence of STRE and HSE motifs in the *pacC* promoter suggests that this gene is regulated by heat shock. As with *gsn* down-regulation under heat stress [5], [6], *pacC* is another example of a gene in *N. crassa* having STRE and HSE binding sites in its 5′-flanking region being down-regulated at the transcriptional level by heat stress. The *gsn* promoter has one STRE motif, which was demonstrated to be involved in the gene transcription modulation when cells were exposed to heat shock (transferred from 30°C to 45°C) [6].

In *S. cerevisiae* the STREs are described as *cis* regulatory motif that mediates the transcriptional activation of genes through the action of the stress response regulator proteins Msn2p/4p [32]. Orthologous proteins have not been identified in the *N. crassa* genome database, suggesting that in this organism STRE may modulate gene expression by an alternative mechanism [33]. The activation of protein kinase A (PKA) leads to Msn2/4p phosphorylation and its cytoplasmic accumulation, thereby preventing the transcription of STRE-regulated genes [34]. Recently, Casado *et al.* [35] demonstrated that the exposure of *S. cerevisiae* to alkaline medium resulted in a decrease in cAMP that led to inhibition of the PKA pathway and the accumulation of Msn2/4p in the nucleus. These data indicate that the adaptative response to alkaline pH involves PKA-regulated Msn2/4p-mediated gene remodeling. Since *N. crassa* lacks Msn2/4p orthologues we hypothesize that the down-regulation of *pacC* under heat shock and alkaline pH results from the activation of proteins that mediate gene transcription regulation under heat stress through STRE. Whether the PKA signaling pathway is involved in this process remains to be demonstrated. We have previously reported that a *N. crassa* mutant strain with an inactive PKA pathway showed hyper-accumulation of glycogen during growth when compared to the wild-type strain [7]. However, the *gsn* transcript level was not increased in this strain; rather, GSN was less phosphorylated *in vitro* and therefore more active, suggesting that GSN post-translational modification is likely to be the main mechanism controlling glycogen accumulation during vegetative growth when the PKA pathway is inactive.

### PACC Binds to the *gsn* Promoter *in vitro* and *in vivo* and is Active at Normal and Alkaline pH

DNA shift experiments showed that truncated PACC having the DNA-binding domain was able to bind to a DNA fragment from the *gsn* promoter containing the PacC binding site. The DNA-binding reaction was specific, based on three criteria: (1) binding was reduced when a DNA fragment mutated in the PacC core sequence was used, (2) a DNA oligonucleotide containing the PacC binding site competed in the binding reactions by reducing the DNA-protein complexes, and (3) anti-PACC antibody led to a DNA-protein supershift. Together, these findings revealed that the PacC binding site in the *gsn* promoter was the target for the binding of recombinant PACC. In our results, it was observed binding reduction when the mutated probe was used, instead of no binding, which was completely abolished in the presence of the DNA oligonucleotide as competitor. We suggested either that the core sequence neighborhoods are important for PACC recognition or the *N. crassa* PACC has an alternative binding site similar to what was described for Rim101p in *C. albicans* [36]. DNA-protein complexes were also observed when crude cellular extracts were used as protein sources. Interestingly, proteins from extracts prepared from wild-type cells grown at normal and alkaline pH were able to bind to the DNA fragment *in vitro* and *in vivo*, and the PACC present in these extracts had the same molecular mass (<60 kD).

According to the model for pH-mediated regulation of gene expression in *A. nidulans* [23] full-length PacC (PacC^72^) is synthesized as an inactive form and an alkaline signal is transduced by the *pal* signaling pathway, thereby activating PacC^72^ to PacC^53^ and PacC^27^ by two successive proteolytic steps. The *S. cerevisiae* Rim101p is similar in size to the *A. nidulans* protein but they differ in that the yeast protein is proteolytically processed at the C-terminus by a single cleavage step and the smaller size form (similar to PacC^27^) has not been detected [16]. The results described here suggest that *N. crassa* PACC is subject to only one proteolytic processing step since the molecular mass of the protein present in cells grown at alkaline pH was lower than the theoretical value of 67 kD. According to Peñas *et al.* [37], *N. crassa* PACC has a conserved protease box that may lead to a processed form of PACC with a molecular mass of ∼55 kD, the size found in our experiments. The most intriguing result concerning PACC was that the forms detected before and after pH stress had the same molecular mass, which indicates that alkaline pH is not the signal for proteolytic processing. Rather, the protein is already processed during growth at pH 5.8.

The finding that *N. crassa* has orthologues to all six Pal proteins, including PalH which, together with PalI, function as membrane pH sensors in *A. nidulans* [23], is particularly interesting. According to the model proposed for *A. nidulans*, the pH signal is transduced to downstream components by PalF phosphorylation and ubiquitination, leading to endocytosis of the PalF/PalH complex [38]. By overexpressing PalF covalently attached to ubiquitin in a null *palH* background Hervás-Aguilar *et al.* [39] have shown that PalF ubiquitination activates the signaling pathway under acidic conditions. The authors concluded that ubiquitination of PalF commands the cascade of intracellular events that mediate fungal adaptation to environmental pH.

Our results clearly demonstrated that in *N. crassa* ambient pH controls glycogen accumulation by regulating *gsn* expression, i.e., by regulating glycogen synthesis and not degradation. Although the data point to a central role for PACC many questions remain unanswered. One major concern is the fact that PACC shows the same molecular mass before and after alkaline pH stress, indicating that if there is any processing it must occur independently of ambient pH. Another question relates to the *in vivo* results showing that endogenous PACC binds to the *gsn* promoter even at pH 5.8. If there is protein association at both pH values, how are the differences in *gsn* expression and glycogen content to be explained? Since *N. crassa* has all six Pal protein orthologues we might speculate that PalH may function as a pH sensor, as in *A. nidulans*, and that all six Pal homologues are active components of the pH signaling pathway. Finally, is PACC the only regulator of glycogen accumulation and *gsn* expression at alkaline pH? Other molecular mechanisms are known to be involved in adaptation to environmental pH in *S. cerevisiae* and *C. albicans* [40]. The results described here reveal interesting differences between *N. crassa* and *A. nidulans* with regard to the pH signaling pathway that deserve to be investigated.

## Materials and Methods

### *Neurospora Crassa* Strains and Growth Conditions

*Neurospora crassa* FGSC#9718 (*mat a, mus-51::bar*), the wild-type background strain used in these experiments, was purchased from the Fungal Genetics Stock Center (FGSC, University of Missouri, Kansas City, MO, USA, http://www.fgsc.net) [41]. A *pacC* knockout mutant strain (*pacC^KO^*) was generated following the knockout procedures described elsewhere [22] based on the strains FGSC#9568 (*mat a, mus-52::hyg*) and FGSC#2490 (*mat A*). All strains were maintained on solid Vogel's minimal (VM) medium, pH 5.8 [42] containing 2% sucrose at 30°C.

Conidia from 10 day cultures of wild-type and *pacC^KO^* strains were suspended in sterile water and counted. For radial growth analysis, 10^7^ conidia/mL were inoculated onto plates containing solid VM medium and VM medium supplemented with yeast extract at pH 4.2, 5.8 and 7.8 for 24 h. Images of colony morphology were captured after 24 h using an AxioCam ICc3 coupled to the stereoscope trinocular Discovery V8 (Zeiss) at 80 x magnification. For linear growth, conidia were inoculated in race tubes containing VM medium at pH 4.2, 5.8 and 7.8 and incubated at room temperature. Measurements were made every 24 h.

For pH stress experiments, 10^9^ conidia/mL were first germinated in 4 L of VM medium (pH 5.8) at 30°C, 250 rpm, for 24 h. After this period, the culture was filtered and the mycelia divided into three samples. One was frozen in liquid nitrogen and stored at −80°C for further processing (control sample, not submitted to stress) while the remaining two samples were transferred into 1 L of fresh VM medium containing 0.5% sucrose at pH 4.2 (for acid stress) and 7.8 (for alkaline stress). Samples (125 mL) from mycelia submitted to both pH conditions were harvested after different periods of incubation. For the experiments combining pH and heat stress, mycelia obtained from the wild-type strain after 24 h were filtered and divided into three samples that were transferred into 1 L of fresh VM medium containing 0.5% sucrose at pH 4.2, 5.8 and 7.8 preheated to 45°C. Samples (125 mL) were harvested after 30 min incubation and processed as before.

In both stress experiment samples from the wild-type strain were subjected to the recuperation conditions. The remaining mycelia from the pH stress experiments were filtered and transferred back into 400 mL of fresh VM medium at pH 5.8 and 30°C. Samples were collected after 30, 60 and 120 min of recuperation. For experiments combining pH and heat stress the remaining mycelia were transferred back into 400 mL of fresh VM medium pre-heated at 30°C at the three different pH. Cells from 120 mL samples were harvested after 30, 60 and 120 min of recuperation and processed. The mycelial samples were used for glycogen quantification and RNA extraction.

### Generation of a *pacC^KO^* Mutant by Gene Replacement

A strain bearing a deletion in the *pacC* gene was generated as described by Ninomiya *et al*. [21]. Briefly, three PCR fragments were produced using the following pairs of primers: fragment 1, primers 0090-F1 and 0090-R2, fragment 2, 0090-F2 and 0090-R3, and fragment 3, primers 0090-F3 and 0090-R1 (Table 1). Fragments 1 and 3 contained a 1.5 kb upstream and downstream of the ORF NCU00090 (*pacC*, 1,866 bp), respectively, with an additional 20 bp overlapping sequence from the *bar* gene. Both fragments were amplified from wild-type strain genomic DNA. Fragment 2 contained the *bar* ORF (1 kb) plus overlapping sequences from the *pacC* gene at both ends and was amplified from the pIV9A-1 plasmid [6]. All three fragments were purified using a Qiagen gel purification kit, and 100 ng of each fragment was mixed and used to amplify a single fragment (4 kb) with the primers 0090-F1 and 0090-R1. All PCRs were done using Phusion High Fidelity *Taq* polymerase (NEB). The final PCR fragment was purified and used to transform 10 day conidia from the strain FGSC#9568. Transformants were selected by plating in medium containing Basta (200 µg/mL). Correct integration of the fragment into the genome was detected by PCR using primers 0090-F1 and 0090-R4 that annealed downstream from the amplified fragment. One transformant containing the right integration was backcrossed with FGSC#2490 to generate a homokaryotic strain.

**Table 1:** Oligonucleotides used in this study.

Primer	Sequencea, b, c	Source	Name	Position^d^
0090-F1	5'-CATGCTCATCCGCCGTCAACC-3'	*pacC* 5'-flanking region	–	−1505 to −1485
0090-R2	5'- CAATATCATCTTCTGTCGACGGTTGCTGCTGTGTGACCG-3'	*pacC* 5'-flanking region	–	−1 to −19
0090-F2	5'- CGGTCACACAGCAGCAACCGTCGACAGAAGATGATATTG-3'	pBARGEM7-2	*bar* ORF	–
0090-R3	5'- GCCTTCCTTACATGGGGAGCGTCGACCTAAATCTCGGT-3'	pBARGEM7-2	*bar* ORF	–
0090-F3	5'- ACCGAGATTTAGGTCGACGCTCCCCATGTAAGGAAGGC-3'	*pacC* 3'-flanking region	–	2042 to 2061
0090-R1	5'- TAGTATGCTGCGCGGCGATAC-3'	*pacC* 3'-flanking region	–	3455 to 3476
0090-R4	5'- GATTGGACAGAAGCAGGGAAG-3'	*pacC* 3'-flanking region	–	3565 to 3585
90-F	5'-CATATGTCGTCCACACCAGCCCAG-3'	NCU00090 ORF	–	1 to 21
90-R2	5'-GGATCC ***TTA***CTTGTGAACTGGAGCCTG-3'	NCU00090 ORF	–	639 to 622
PacC-F	5'-GACCCAACAGCCCAACTT-3'	*gsn* promoter	*pacC* probe	1918 to −1901
GSN-RP3	5'-GCAACGAATACTCCCATG-3'	*gsn* promoter	*pacC* probe	1773 to −1790
mPacC-F	5'-GCCGTCTTTGGGCCAG**AAATTA**CGTGAGATCGGGCCCGC-3'	*gsn* promoter	m*pacC* probe	−1828 to −1790
mPacC-R	5'-GCGGGCCCGATCTCACG**TAATTT**CTGGCCCAAAGACGGC-3'	*gsn* promoter	m*pacC* probe	−1790 to −1826
pGSN-F	5'-TTGGAAAGACGGGACC-3'	*gsn* promoter	m*pacC* probe	−2386 to −2370
GSN-RP2	5'-CTGTTGACCCTGCGTTACG-3'	*gsn* promoter	m*pacC* probe	−1269 to −1286
HSE-F	5'-GGGGAATTTGTGGCTGA-3'	*gsn* promoter	–	−2087 to −2071
OligoPacC-F	5'-CTTTGGGCCAGCTTGGCCGTGAGATCG-3'	*gsn* promoter	oligo *pacC*	−1823 to −1797
OligoPacC-R	5'-CGATCTCACGGCCAAGCTGGCCCAAAG-3'	*gsn* promoter	oligo *pacC*	−1797 to −1823

a The *Nde*I and *Bam*HI restriction sites are underlined in the 90-F and 90-R2 sequences, respectively.

b The TAA stop codon inserted in the ORF NCU00090 sequence is represented in bold italics.

c The nucleotides mutated in the PacC motif are represented in bold.

d Primer positions are in relation to the ATG start codon.

### *pacC* cDNA Cloning and Production and Purification of the Recombinant Protein

The *pacC* gene encodes a 621 amino acid protein with a theoretical molecular mass of 67 kD. A *pacC* cDNA fragment (639 bp) encoding the N-terminal region containing the zinc finger C_2_H_2_ DNA-binding domain was amplified from the cDNA plasmid library pYADE5 [43] with the oligonucleotides 90-F and 90-R2 (Table 1), as previously described [8]. For expression of the His-ΔPACC recombinant protein the *E. coli* BL21(DE3) pLysS strain harboring the pET-ΔPACC plasmid was used. A truncated protein encompassing amino acids 1 to 213 fused to an N-terminal His_6_-tag was produced. Cells were grown at 37°C in 1 L of LB medium to an OD_600_ of 0.7 and induced with IPTG (final concentration 0.4 mM) for 4 h at 37°C. The cells were harvested by centrifugation, suspended in buffer A (50 mM Tris-HCl, pH 8.0, 300 mM NaCl, 20 mM imidazole, 10% v/v glycerol, 1 mM DTT, 0.5 mM PMSF, 25 mM benzamidine and 5 µg/mL each of antipain, leupeptin and pepstatin A) containing 0.5% Triton X-100 and 0.5% Tween 20 and lysed by sonication (5 cycles of 30 s sonication and 30 s on ice). After centrifugation, the supernatant was subjected to affinity chromatography on a HisTrap HP column (GE Healthcare) using an ÄKTA Prime purification system. The recombinant protein was eluted with a linear gradient of imidazole (20–500 mM) in buffer A and dialyzed two times against 1 L of dialysis buffer (10 mM Tris-HCl, pH 7.9, 100 mM KCl, 10% v/v glycerol, 1 mM EDTA and 0.5 mM DTT). The purified protein was analyzed by SDS-PAGE followed by Coomassie Brilliant Blue staining [44] and was quantified by the Hartree [45] method using BSA as standard. The purified His-ΔPacC protein was used to raise antibodies in rabbits.

### Glycogen Quantification and Gene Expression Assays

Mycelial pads were ground to a fine powder in a pre-chilled mortar in liquid nitrogen and extracted with lysis buffer (50 mM Tris-HCl, pH 7.6, 100 mM NaF, 1 mM EDTA, 1 mM PMSF, 0.1 mM TCLK, 1 mM benzamidine and 1 µg/mL each of pepstatin and aprotinin). Cell extracts were clarified by centrifugation (3,000×*g*, 10 min, 4°C) and the supernatants were used for glycogen and protein quantification. Glycogen was extracted by digestion with amyloglucosidase (30 mg/mL) and α-amylase (10 mg/mL) after ethanol precipitation, as previously described [7]. Free glucose was measured with a glucose oxidase kit and the glycogen content was normalized to the total protein concentration. Total protein was quantified by the Hartree method [45].

For gene expression analysis by Northern blotting, total RNA was extracted with LiCl [46] from mycelial samples subjected to pH and combined pH and temperature stress. Total RNA (15 µg) was electrophoresed on a 1.2% agarose-formaldehyde denaturing gel [47] at 65 V for 5 h and then transferred to neutral nylon membranes (Hybond-N, GE Healthcare) in 2 x SSC. The blots were probed either with a 678 bp *gsn* cDNA, or 798 bp *gpn* cDNA, or 639 bp *pacC* cDNA fragments (10^6^–10^8^ cpm) radiolabelled with α-^32^P-dATP (3,000 µCi/mmol) by random priming (NEBlot kit, Biolabs) in 5–10 mL of ULTRAhyb hybridization solution (Ambion) at 42°C overnight. After hybridization, the blot was washed twice in 2 x SSC containing 0.1% SDS for 20 min, and twice in 0.1 x SSC containing 0.1% SDS for 20 min followed by exposure to an X-ray film.

The *pacC* gene was also analyzed at protein level by Western blot. Total proteins from cells of the wild-type strain cultured at pH 5.8 for 24 h (control) and that subjected to alkaline pH stress (pH 7.8) for 1, 2, 3 and 4 h were prepared as described for glycogen extraction using glass beads. Proteins (100 µg) were separated by 12% SDS-PAGE gels [44] and electrophoretically transferred to a nitrocellulose membrane. Immunoblotting was performed with polyclonal anti-PACC antibody and band intensities were normalized to parallel blots probed with anti-α-tubulin antibody (Sigma). Blots were subsequently probed with HRP-conjugated secondary antibodies (Sigma) and developed with luminol reagent.

### Electrophoretic Mobility Shift Assay (EMSA)

DNA-protein binding reactions were carried out in 30–80 µL of 1 x binding buffer (25 mM HEPES-KOH, pH 7.9, 20 mM KCl, 10% v/v glycerol, 1 mM DTT, 0.2 mM EDTA, 0.5 mM PMSF, 12.5 mM benzamidine and 5 µg/mL each of antipain and pepstatin A) containing 2 µg of poly(dI-dC).(dI-dC) as a non-specific competitor and 0.5–5 µg of His-ΔPACC recombinant protein. In some experiments, 35 µg of protein from chromatographic fractions (obtained as described below) was used. A radiolabeled DNA probe (∼10^4 ^cpm) was added and the reactions were incubated at room temperature for 20 min. Free probe was separated from DNA-protein complexes by electrophoresis on a native 5% polyacrylamide gel in 0.5 x·TBE buffer at 300 V, 10 mA and 10°C. After electrophoresis, the gel was dried and autoradiographed. For competition assays, an excess of specific DNA competitor was added to the binding reactions 10 min prior to incubation with the radiolabeled probe.

For the supershift experiments, 5 µg of recombinant His-ΔPACC protein was incubated with an excess of polyclonal anti-PACC antibody (title 1∶500) for 10 min on ice prior to the addition of radiolabeled *pacC* probe and further 20 min incubation at room temperature.

### Preparation and Fractionation of Crude Cellular Extract for EMSA

Cells from the wild-type and *pacC^KO^* strains exposed to alkaline stress (pH 7.8, 60 min) or not (pH 5.8, control) were used to prepare crude cellular extracts. About 10 mg of frozen mycelia were ground to a fine powder in liquid nitrogen in a pre-chilled mortar, homogenized in 20 mL of lysis buffer (15 mM HEPES-KOH, pH 7.9, 10% v/v glycerol, 500 mM KCl, 5 mM MgCl_2_, 0.5 mM EDTA, 1 mM DTT, 0.5 mM PMSF, 25 mM benzamidine, 50 mM NaF and 10 µg/mL each of antipain and pepstatin A) and stirred with glass beads in eight cycles that consisted of 30 s of stirring and 30 s on ice. Crude cellular extract was recovered after centrifugation (3,200 × *g*, 2 min, 4°C), dialyzed against buffer D (15 mM HEPES-KOH, pH 7.9, 15% v/v glycerol, 100 mM KCl, 1 mM EDTA) at 4°C for 2 h, and cleared by centrifugation (20,000 × *g*, 20 min, 4°C) before loading onto a Heparin-Sepharose FF column (GE Healthcare). The proteins were eluted with a 0.1–1.5 mM KCl linear gradient and the protein fractions were dialyzed against buffer D containing 0.5 mM PMSF, 25 mM benzamidine and 50 mM NaF and then frozen in liquid nitrogen and storage at −80°C. Total protein was quantified by the Hartree method [45]. Proteins from the chromatographic fractions (35 µg of total protein) were analyzed for their ability to bind to the DNA fragment of the *gsn* promoter containing the *pacC* binding site.

### DNA Probe and Competitors for EMSA

A putative *cis* PacC motif (5′-GCCAAG-3′) was identified in the *gsn* gene 5'-flanking region (starting at nucleotide −1807) by analysis *in silico* using the MatInspector tool (www.genomatix.de). To produce the *pacC* probe, a 146 bp DNA fragment containing the PacC motif was amplified from the pIV9A-1 plasmid (GenBank#AF417205) using oligonucleotides PacC-F and GSN-RP3 (Table 1) in the presence of α-^32^P-dATP (3,000 Ci/mmol) and purified on 2% low-melting point agarose gel. A mutant probe (m*pacC*) was prepared by changing the element core sequence from 5′-CTTGGC-3′ to 5′-AAATTA-3′ by site-directed mutagenesis in a two-step PCR [48]. The oligonucleotide pairs mPacC-F/GSN-RP2 and pGSN-F/mPacC-R (Table 1) were used in the first reaction to amplify two fragments, and the oligonucleotide pair pGSN-F/GNS-RP2 was used in a second reaction to amplify the whole DNA fragment containing the mutation. The 311 bp fragment was subcloned into the vector pMOS-Blue (GE Healthcare) and the desired mutation was confirmed by DNA sequencing. For EMSA, the mutant probe (m*pacC*) was amplified by PCR in the presence of α-^32^P-dATP using the oligonucleotides PacC-F and GSN-RP3 and then purified.

The unlabeled 146 bp *pacC* probe was used as a specific DNA competitor. A 27 bp DNA oligonucleotide was also used as a competitor after annealing the complementary oligonucleotides OligoPacC-F and OligoPacC-R (Table 1). The specific competitors were quantified by measuring the absorbance at 260 nm and then added to the binding reaction in a 15- to 40-fold molar excess for the specific competitor and a 1- to 15-fold molar excess for the DNA oligonucleotide.

### ChIP-PCR Analysis

ChIP assays were performed according to Tamaru *et al*. [49], with modifications. Briefly, the wild-type strain was grown in liquid VM medium at 30°C, 250 rpm, for 24 h, and subjected to pH stress as previously described. Mycelial samples (125 mL aliquots) were subsequently transferred into 500 mL flasks and the chromatin was fixed by adding formaldehyde to 1% final concentration followed by incubation for 30 min at 30°C and 250 rpm. Formaldehyde was quenched by adding 125 mM glycine to each sample and then incubating at 30°C, 250 rpm, for 10 min. Chromatin prepared from each sample was pre-cleared with normal rabbit IgG (Upstate) and protein A agarose (Sigma) 50% slurry pre-blocked with sonicated salmon-sperm DNA and then immunoprecipitated with polyclonal anti-PACC antibody and protein A-agarose. As a negative control, a reaction without anti-PACC antibody was done. The 146 bp DNA fragment associated with the PACC protein was amplified by PCR using the oligonucleotides PacC-F and GSN-RP3 and the reaction products were analyzed on a 2% agarose gel. A plasmid containing the entire sequence of the *gsn* gene, including its 5′- and 3′-flanking regions, was used as a positive control for PCR.
